# Population dynamic of the extinct European aurochs: genetic evidence of a north-south differentiation pattern and no evidence of post-glacial expansion

**DOI:** 10.1186/1471-2148-10-83

**Published:** 2010-03-26

**Authors:** Stefano Mona, Giulio Catalano, Martina Lari, Greger Larson, Paolo Boscato, Antonella Casoli, Luca Sineo, Carolina Di Patti, Elena Pecchioli, David Caramelli, Giorgio Bertorelle

**Affiliations:** 1 Dipartimento di Biologia ed Evoluzione, Università di Ferrara, Ferrara, Italy; 2 Dipartimento di Biologia Evoluzionistica, Università di Firenze, Firenze, Italy; 3 Department of Archaeology, Durham University, Durham, UK; 4 Dipartimento di Scienze Ambientali, Università di Siena, Siena, Italy; 5 Dipartimento di Chimica Generale e Inorganica, Chimica Analitica, Chimica Fisica, Università di Parma, Parma, Italy; 6 Dipartimento di Biologia Animale, Università di Palermo, Palermo, Italy; 7 Museo di Geologia e Paleontologia G Gemellaro, Università di Palermo, Palermo, Italy; 8 IASMA Research and Innovation Centre, Fondazione Edmund Mach, Trento, Italy

## Abstract

**Background:**

The aurochs (*Bos primigenius*) was a large bovine that ranged over almost the entirety of the Eurasian continent and North Africa. It is the wild ancestor of the modern cattle (*Bos taurus*), and went extinct in 1627 probably as a consequence of human hunting and the progressive reduction of its habitat. To investigate in detail the genetic history of this species and to compare the population dynamics in different European areas, we analysed *Bos primigenius *remains from various sites across Italy.

**Results:**

Fourteen samples provided ancient DNA fragments from the mitochondrial hypervariable region. Our data, jointly analysed with previously published sequences, support the view that Italian aurochsen were genetically similar to modern bovine breeds, but very different from northern/central European aurochsen. Bayesian analyses and coalescent simulations indicate that the genetic variation pattern in both Italian and northern/central European aurochsen is compatible with demographic stability after the last glaciation. We provide evidence that signatures of population expansion can erroneously arise in stable aurochsen populations when the different ages of the samples are not taken into account.

**Conclusions:**

Distinct groups of aurochsen probably inhabited Italy and northern/central Europe after the last glaciation, respectively. On the contrary, Italian and Fertile Crescent aurochsen likely shared several mtDNA sequences, now common in modern breeds. We argue that a certain level of genetic homogeneity characterized aurochs populations in Southern Europe and the Middle East, and also that post-glacial recolonization of northern and central Europe advanced, without major demographic expansions, from eastern, and not southern, refugia.

## Background

Ancient DNA offers the unique opportunity to broaden the time depth of population genetic analyses. Moreover, it is the only tool capable of deciphering the demographic histories of extinct species [1-4]. The aurochs, *Bos primigenius*, was one of two wild bovine species in Europe (*Bison bonasus *was the other). Probably as a consequence of human hunting and habitat reduction [5], the aurochs went extinct in 1627, when the last individual died in Poland. This species most likely originated in India between 1.5 and 2 MYA ago and later occupied many parts of Asia, North Africa and Europe. The first documented remains in Europe dates back to 275,000 years ago [5]. Its demographic history is largely unknown and, in particular, it is unclear to what extent it was shaped by Pleistocene climate changes. Indeed, the genetic structure and population history of many European species were largely influenced by the alternating of glacial and inter-glacial periods [3,6-8].

When modern samples are analysed, refugial areas generally harbour greater genetic diversity and multiple phylogenetic clusters with deeply rooted branches that indicate their antiquity beyond the most recent Ice Age; conversely, if we exclude the contact zones where distinct migratory routes mix [9], non-refugial areas display reduced diversity and show only a subset of the phylogenetic clusters present in the refugia [7]. Based mainly on genetic evidence of this type, four models have been proposed to describe the post-glacial recolonization routes in Europe [7,10,11]. The models differ in the relative contribution of the refugia populations to the expansion process, and the genetic data analysed for several species seemed always to fit the predictions of at least one of these models. Recently, however, a growing number of studies reports unexpected patterns of diversity [e.g., [12-14], and even within refugia, the genetic structure might have been much more complex than previously acknowledged [15]. Recent theoretical studies (reviewed in [16]) also suggest that a range expansion from a single refugium can lead to a geographically structured population, which can be easily misinterpreted as the result of an expansion from multiple distinct refugia. Therefore, the geographic distribution of genetic clusters might not always be informative for identifying recolonization routes.

The analysis of DNA from ancient samples represents a key step to overcome these problems. It opens a genetic-variation window directly onto the past, useful to investigate ancient population dynamics and to compare the demographic history of refugial and non-refugial populations [3,17]. In particular, ancient DNA is a fundamental tool available to investigate aurochs dynamics. In fact, the aurochs surviving descendents, cattle, have a complex history of selection and displacement which heavily interferes with any attempt to reconstruct past events that occurred in the wild.

DNA data from ancient aurochs specimens can also be informative for the reconstruction of the domestication process (e.g., [18,19]). Recent archaeological evidence suggests that taurine cattle (*Bos taurus*) were initially domesticated in the upper Euphrates Valley around10,000-11,000 years B.P. [20] This *single origin *hypothesis implies that present day European breeds, which belong to the taurine type, all descended from Fertile Crescent ancestors. The phylogenetic dichotomy between northern and central European *Bos primigenius *(P and E) and *Bos taurus *(T) mtDNA clades was interpreted as a clear evidence supporting the single origin hypothesis [19,21,22]. But five Italian pre-domesticate aurochsen with T haplotypes [18], and a P haplotype recently found in one Korean cow generically classified as "beef-cattle" [23] raise doubts about the *primigenius-taurus *mtDNA distinctiveness, and, as a consequence, about the single origin hypothesis. As suggested also by the large genetic diversity in the Italian, compared to other European breeds [18,23-26], the domestication process in Europe, or at least in Italy, requires a careful reconsideration.

For this study we generated partial mtDNA control region sequences from 14 ancient aurochsen samples collected from a geographically diverse range of locations across Italy. Samples from Southern refugia are particularly valuable since DNA preservation is uncommon in warmer climates [27]. Indeed, several aurochs sequences have been analyzed in northern and central Europe [19,21,22] but only few sequences have been described so far from Southern areas [18,22,28]. The main goals of our study were: i) to investigate the genetic variability and the demographic history of Italian aurochsen, especially in comparison with the northern and central European groups (i.e., comparing a refugia vs. a non-refugia population); ii) to study whether and how the analysis of aurochs data collected at different time intervals (heterochronous sampling) may produce erroneous conclusions when simple statistics which assume simultaneous (isochronous) sampling are used; and iii) to test previous insights suggesting a role for the Palaeolithic aurochs in the domestication process in Italy [18].

## Results

### aDNA laboratory results

The values of amino acid racemization obtained in all our samples suggested good biochemical preservation (< 0.10 for aspartic acid). This result appears compatible with DNA survival [29,30], although some concern have been raised on the utility of this approach [31,32]. The alanine D/L and glutamine D/L are lower compared to the aspartic acid D/L, which is expected since the racemization of alanine and glutamine is slower than that of aspartic acid. This difference suggests that modern (contaminant) aminoacids are absent [30]. Sporadic contamination is unlikely when the number of molecules that PCR will use as template (or target DNA) is greater than 1,000 [30]. By using real-time PCR, we quantified the number of DNA molecules in the extracts from 20 samples, selected to be representative of different Palaeolithic and Late Mesolithic sites (Table S1, see Additional file 1); we found well preserved DNA in 18 of the 20 samples, with numbers of DNA molecules varying from 1,000 to more than 5,000 (Table S1, see Additional file 1). After positive extraction and amplification of 14 samples, and cloning of PCR products for all them, we obtained the entire control region sequence for one sample; 13 other samples yielded partial sequences from at least one of the three fragments in which HVR-I had been subdivided (Table S2, see Additional file 2). Independent replication of extraction, amplification of the highly variable first fragment (134 bp), and cloning was performed at CEA (Trento) ancient DNA laboratory for seven bone samples (Table S2, see Additional file 2). The replicated sequences matched those obtained in the Florence laboratory (see Table S2, see Additional file 2). Only sporadic G-to-A change and C-to-T change were observed in some clones, likely due to post-mortem cytosine deamination [33]. The Taq misincorporation value for all 202 cloned sequences was estimated to be 1.21 substitutions, and 87% of the clones possessed the consensus sequence. These results suggest that the DNA templates were not significantly damaged.

### Analysis of haplotype diversity

On the basis of the diagnostic sites identified in previous studies [19,22,25], 12 sequences were assigned to the T haplogroups and two sequences belong to the P haplogroup (Table S3, see Additional file 3). In other words, the vast majority of the Italian aurochsen analysed in this study showed the same type of mtDNA sequence observed previously in 5 Italian aurochsen [18] and in all the extant European cattle breeds analysed so far, though two individuals share the same haplogroup with the aurochsen from northern and central Europe (and with a single cattle sequenced in Korea generically classified as "beef cattle" [23]). All the DNA sequences are available from GenBank under the accession numbers from GU434117 to GU434130.

**Figure 1:**
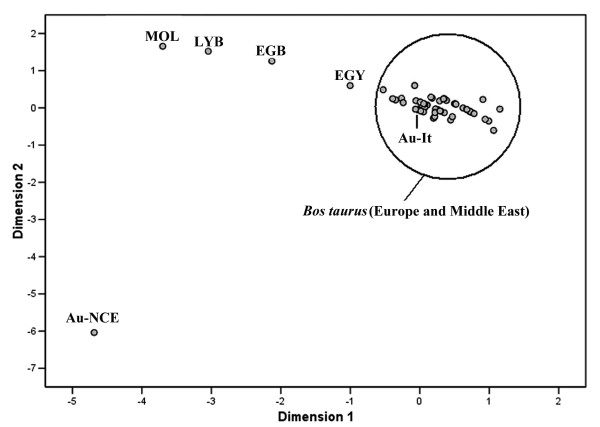
**Two-dimensional MDS plot based on Φ*st *distances between aurochs population and 53 taurine cattle breeds; stress value = 0.053**. Au-NCE = Northern and central European aurochs; Au-It = Italian aurochs; MOL (Morocco Olmez), LYB (Lybian Local), EGB (Egyptian Baladi) and EGY (Egypt) are all cattle breeds from Africa. The complete list of the cattle breeds used for this figure is reported in Table S4 (see Additional file 4).

The dataset was then reduced to ten sequences using an overlapping fragment of 120 bp from position 16042 to 16161. These sequences were jointly analysed with 55 previously published aurochs sequences from Italy and northern/central Europe [18,22], and 800 modern cattle representing 53 breeds from Europe, Turkey, Middle East and North Africa (the list of breeds and the sample sizes are available in Table S4, see Additional file 4). In this data set, the group of Italian aurochsen (hereafter, Au-It) and the group of northern and central European aurochsen (hereafter, Au-NCE) were composed of 15 and 50 individuals respectively. Even though they are derived from different geographical areas, the Au-NCE group included specimens with highly similar sequences. In fact, when Au-NCE was split into three geographic groups (Britain, east central Europe, and west central Europe), no significant genetic divergence was found (AMOVA: Φ*st *= 0.02, P = 0.16). The Φ*st *genetic distances between the two groups of aurochsen and the modern breeds were graphically represented by a MDS analysis (Figure 1).

As observed in a previous study based only on five Italian aurochsen [18], the Italian *Bos primigenius *fell into the cluster that included all modern breeds in Europe. North African breeds, due to the prevalence of T1 haplotypes in this region, appear related, but clearly distinct from European breeds. Conversely, the *Bos primigenius *specimens excavated outside Italy, joined here in the Au-NCE group, and all characterized by P sequences, are distantly related to all the other modern or ancient samples. The Φ*st *value between the two ancient groups (Au-It *vs*. Au-NCE) was very high (0.727, *P *< 0.0001), which is consistent with their haplogroup frequencies. However, this value needs to be viewed with caution since the two groups both included samples with different ages; nevertheless, these results clearly suggest the existence of a strong north-south geographic structure in this species before domestication, with very limited gene flow across the Alps.

**Figure 2:**
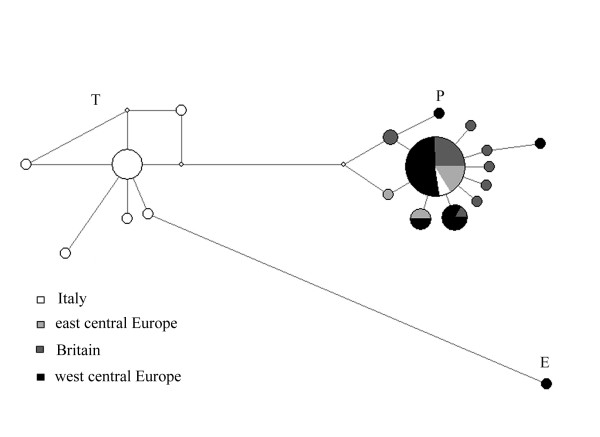
**Median-joining network of the 120 bp aurochs haplotypes**. The branch length is proportional to the number of substitutions; the node diameter is proportional to the haplotype frequency. The names of the major haplogroups are shown.

The phylogeographic structure of the aurochs haplotypes (see Figure 2, where Au-NCE is subdivided as before into geographic areas) was characterized by three major clades: 1) T sequences only found in Italy, 2) P sequences separated among them by no more than two mutations and distributed throughout the European regions (including Italy), and 3) a highly divergent E group found only in one German Neolithic sample [22]. These results, which are in agreement with previous analyses based on longer mtDNA fragments [22], indicate that the 120 bp fragment used in our data-set contains enough information to discriminate between these clades.

Considering the P group of sequences as representative of the genetic variation in northern and central Europe (thus excluding the divergent E sequence), a star-like pattern suggesting a demographic increase in these area was identified in previous studies [21,22]. The phylogenetic structure in Italy is clearly different: the highly divergent T and P clades, typical of modern breeds and northern and central European aurochs populations, respectively, are both observed in pre-Neolithic samples.

### Choice of the best fitting demographic model

The demographic history of *Bos primigenius *in Italy and northern/central Europe, and the possible differences in these two areas, was further analysed using BEAST under two models: constant population size and exponential change. This analysis was performed separately for the Au-It and the Au-NCE groups.

The Au-NCE data set was reduced to the 37 sequences published and analysed by [22] so that the sequences retained their full 360 bp fragment size, and we could also compare our results with those in [22]. Using the natural logarithmic scale of Jeffreys to interpret the Bayes Factor (as reported in [34]), we found "positive" evidence in favour of the exponential change model (BF = 3.7). The median value of the posterior distribution of the growth rate, *g*, was small and negative (around -2*10^-5^), i.e. compatible with a limited population contraction. However, the 95% high posterior density, though clearly left-shifted, included zero (Table 1) suggesting that demographic stability could not be excluded. In other words, in contrast with the analysis reported in [22], the data for the northern and central European aurochs appeared inconsistent with a population expansion: a demographic model with either population size stability or a very limited decline seems more compatible with the genetic variation pattern.

**Table 1:** Demographic parameters estimated by BEAST under the a priori model of exponential growth.

	Ne	TMRCA_P_	TMRCA_T_	TMRCA_Total_	g
**Au-It**	3.3 (0.1 ÷ 12.0)	6.0 (4.3 ÷ 11.6)	24.0 (11.0 ÷ 59.0)	44.0 (11.0 ÷ 130.0)	0.1 (-0.7 ÷ 0.9)

**Au-NCE**	1.7 (0.28 ÷ 4.0)	18.0 (11.0 ÷ 32.0)	-	57.0 (21.0 ÷ 114.0)	-2.17 (-9.5 ÷ 3.3)

Median values and 95% high posterior density (in parenthesis) are shown. *Ne*, the effective population size, is given expressed in thousands of individuals. TMRCA_P_, TMRCA_T_, and TMRCA_Total _are the estimated times (in thousands of years) to most recent common ancestor for haplogrup P, haplogroup T, and the total sample, respectively. The growth rate, *g*, is reported in 10^-5 ^units per generation. Au-It and Au-NCE indicate the Italian and the northern/central European aurochsen, respectively.

When the same analysis was applied to the Italian aurochs (Au-It data set), the Bayes Factor was much smaller (1.6 in favour of the exponential change model, "not worth more than a bare mention" following the Jeffreys natural logarithmic scale), as well as the estimated growth rate (see Table 1). A demographic model with constant population size can therefore explain the Italian aurochs data set, and Au-It showed a long-term effective population size twice as large as Au-NCE (Table 1; population sizes are estimated assuming a generation time of 7 years [35]).

### Simulating the effects of heterochronous sampling

**Figure 3:**
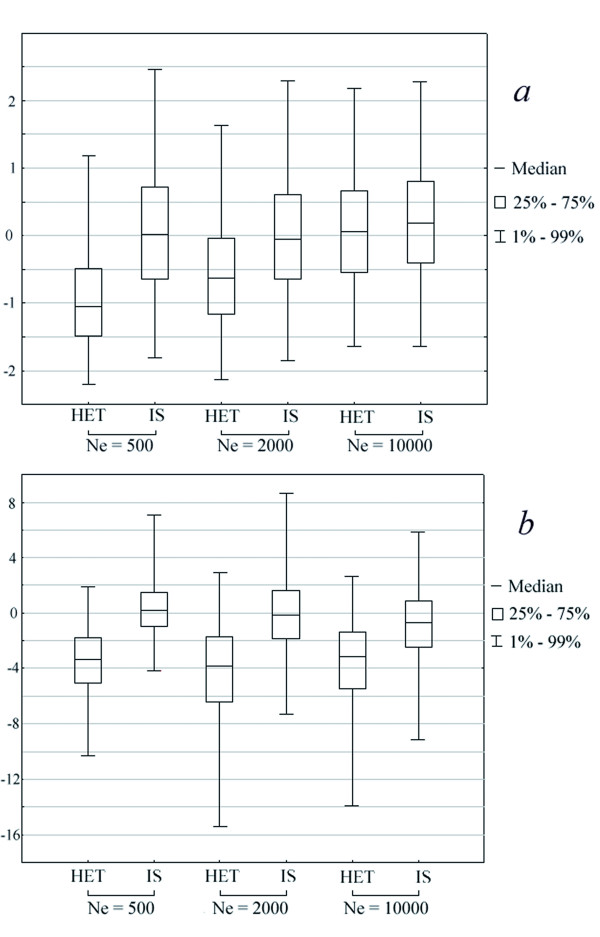
**Box-plot of Tajima's D (a) and Fu's Fs (b) obtained from 1,000 simulations under a constant population size model with various Ne and assuming either an heterochronous (HET) or an isochronous (IS) sampling scheme (see text for details)**.

The BEAST results contrast with both the very similar analysis of the Au-NCE data set by [22], and with the common assumption that the star-like haplotype network in Au-NCE indicates a population expansion. We will discuss the first point in the next section, after we first report the results of our investigation of the second point using a simulation approach. Specifically, we wanted to understand how readily a star-like haplotype network could be interpreted as evidence for a population expansion when the samples are heterochronous. The empirical distributions of Tajima's *D *[36] and Fu's *Fs *[37] were used as indicators for the network shape. The expectation of both these statistics, assuming an isochronous sampling, is zero under a constant population size model, but becomes negative under a population expansion model which also produces a star-like genealogy. The effect of pooling DNA data with different ages (heterochronous sampling) on these expectations seems to depend strictly on details of the temporal sampling scheme[38]. The results we obtained when the samples ages mirror the aurochs data set reported in [22] clearly indicate that the distribution of Tajima's *D *and Fu's *Fs *are skewed towards negative values not only when a demographic expansion is simulated, but also when the population size is constant (see Table 2 and Figure 3).

**Table 2:** Simulation results, based on 1,000 replicates for each scenario (sample size is fixed to 37 individuals, as in the real Au-NCE sample).

Sampling scheme^a^	Ne	g	Statistic^b^	Mean (95% CI)	%N^c^	%P^c^
HET	500	0	*D*	-0.94 (-2.11 ÷ 0.70)	14.5	0.1
			*Fs*	-3.60 (-9.17 ÷ 0.82)	41.9	0.0
IS	500	0	*D*	0.07 (-1.64 ÷ 2.04)	1.9	4.4
			*Fs*	0.30 (-3.18 ÷ 5.09)	4.6	1.4
HET	500	0.001	*D*	-0.93 (-2.10 ÷ 0.87)	15.0	0.1
			*Fs*	-3.55 (-9.57 ÷ 1.35)	40.6	0.0
IS	500	0.001	*D*	0.23 (-1.52 ÷ 2.36)	0.2	6.4
			*Fs*	0.81 (-2.53 ÷ 5.78)	2.1	2.2
HET	500	0.01	*D*	-0.69 (-1.89 ÷ 1.16)	10.5	1.7
			*Fs*	-1.14 (-3.89 ÷ 1.68)	17.1	0.0
IS	500	0.01	*D*	-0.29 (-1.51 ÷ 1.65)	1.4	1.5
			*Fs*	-0.39 (-2.80 ÷ 2.05)	9.8	0.1
HET	2000	0	*D*	-0.54 (-1.93 ÷ 1.27)	8.0	0.5
			*Fs*	-4.24 (-12.81 ÷ 1.54)	29.0	0.0
IS	2000	0	*D*	0.03 (-1.66 ÷ 1.86)	1.8	3.3
			*Fs*	-0.09 (-6.40 ÷ 6.55)	4.6	2.4
HET	2000	0.001	*D*	-1.09 (-2.17 ÷ 0.53)	20.0	0.1
			*Fs*	-4.52 (-10.99 ÷ 0.17)	51.6	0.0
IS	2000	0.001	*D*	-0.53 (-1.76 ÷ 0.96)	4.6	0.1
			*Fs*	-2.25 (-7.74 ÷ 2.05)	18.2	0.0
HET	2000	0.01	*D*	-0.69 (-1.88 ÷ 1.17)	12.0	0.2
			*Fs*	-1.16 (-4.13 ÷ 1.66)	19.9	0.0
IS	2000	0.01	*D*	-1.07 (-2.01 ÷ 0.48)	17.5	0.2
			*Fs*	-2.96 (-7.20 ÷ 0.28)	48.4	0.0
HET	10000	0	*D*	0.04 (-1.47 ÷ 1.83)	1.6	2.9
			*Fs*	-3.79 (-11.83 ÷ 1.46)	17.1	0.0
IS	10000	0	*D*	0.17 (-1.34 ÷ 1.86)	0.3	4.3
			*Fs*	-1.05 (-7.38 ÷ 4.13)	3.3	1.0
HET	10000	0.001	*D*	-1.23 (-2.06 ÷ -0.15)	20.0	0.0
			*Fs*	-9.98 (-20.37 ÷ -2.62)	81.7	0.0
IS	10000	0.001	*D*	-1.18 (-1.92 ÷ -0.23)	11.0	0.0
			*Fs*	-9.40 (-18.83 ÷ -2.58)	75.5	0.0
HET	10000	0.01	*D*	-0.63 (-1.88 ÷ 1.28)	11.6	0.3
			*Fs*	-1.09 (-4.04 ÷ 1.45)	18.1	0.0
IS	10000	0.01	*D*	-1.70 (-2.31 ÷ -0.77)	58.8	0.0
			*Fs*	-8.43 (-16.01 ÷ -2.56)	94.3	0.0

^a ^HET indicates an heterochronous sampling scheme with sequences ages as in Edwards et al. (2007). IS indicates an isochronous sampling scheme, i.e. all sequences have the same age.

^b ^D and Fs correspond to the neutrality statistics developed by Tajima (1989) and Fu (1997), respectively.

^c ^%N and %P indicate the percentage of simulation displaying a negative and positive significant value of the statistics, respectively.

Under constant population size, therefore, an artificial signal of population expansion is likely to arise when the individuals are not sampled from the same time period. The relationship between the fraction of negative and significant *D *and *Fs *values and the different parameters of the simulations is complex and requires more systematic investigations (as in a recent publication [38])). It seems, however, that in all cases where isochronous sampling produces small fractions of significant statistics (i.e., for g = 0 or g = 0.001), the fraction of significant statistics produced by a heterochronous sampling is several times larger.

**Figure 4:**
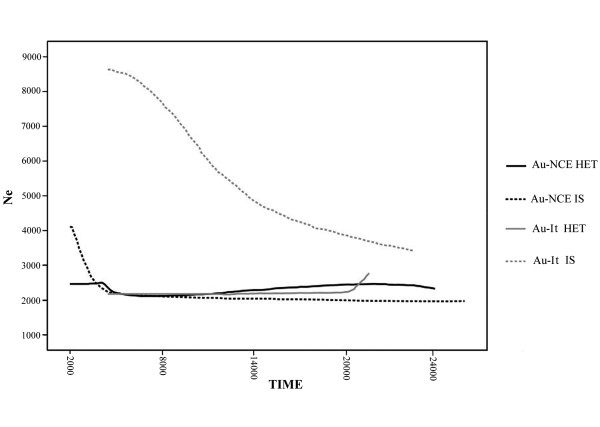
**Bayesian skyline plot of the effective population sizes through time for Au-It (Au-It HET) and Au-NCE (Au-NCE HET)**. The same groups were analyzed assuming an isochronous sampling at 7,000 and 2,000 years B.P. respectively (Au-It IS and Au-NCE IS; see text for details); only the median values are shown.

We have so far demonstrated that an expansion signal can be generated by heterochronous sampling when the temporal information is not taken into account, and that coalescent analyses of both Au-It and Au-NCE were suggestive of a constant, rather than an expanding, population. To further explore this issue, we performed a Bayesian skyline analysis for both aurochs populations using the ages of the sequences as temporal information. We then compared the reconstructed skylines with those estimated assuming that the ancient sequences were sampled simultaneously (isochronous sampling). For clarity of comparison, the isochronous skylines were depicted assuming that the sequences were all sampled at 2,000 and 7,000 years B.P. for Au-NCE and Au-It respectively (i.e., the ages of the most recent sample). The temporal dynamic of the median values is shown in Figure 4. Neither Au-NCE nor Au-It showed evidence of population size changes. However, a strong signal of population expansion emerged when the same groups were considered to be composed by isochronous samples (Figure 4). The 95% highest posterior density (not shown in Figure 4) for all the four analyses was wide, indicating that the results were not very informative regarding the absolute estimate of *Ne*. However, the 95% limits followed the same trend of the median, suggesting that the overall temporal pattern of *Ne *was robust.

## Discussion

Over the last decade, the study of ancient DNA has proven to be particularly helpful in deciphering the evolutionary history of populations [39-42], often revealing unexpected demographic dynamics [2-4,14,43-45]. In this study we investigated the mitochondrial genetic variability of the extinct aurochs.

The distributions of mtDNA haplotypes in Italy and northern/central European areas are very different. Sequences belonging to the P clade, typical of northern and central Europe and not observed in five Italian specimens analysed by [18] were found only in two of the additional 14 sequences here produced. Overall, the vast majority of the Italian aurochsen analysed so far (17 out of 19) have mtDNA sequences belonging to the T clade, which is the phylogenetic group of sequences observed in virtually all European cattle breeds.

The genetic similarity between the Italian aurochs and the European cattle breeds may have important implication for the study and the understanding of the domestication process in this species. Interpretations of the genetic data over the last 15 years, including two recent publications [26,28], have supported both single and multiple origin narratives. It seems, however, that these two hypotheses are based on extreme, oversimplified models developed when wild and domestic status could be determined by mtDNA sequences. Both the process and the data are more complex. European wild and domestic forms, which coexisted at least since the beginning of the Linearbandkeramik culture, around 7,500 years ago [46], shared many genotypic as well as phenotypic traits, and secondary domestication or introgression events possibly occurred in some areas but not in others.

The remarkable difference between the patterns of aurochs genetic variation in Italy and in northern/central Europe is one of the two main results of our study, and confirms the conclusions of a previous study based on only five Italian aurochsen [18]. The other is the lack of demographic size fluctuations in this species in both geographical regions. This latter result conflicts with the conclusions of two previous studies [22,47] which supported a demographic, possibly postglacial, expansion in northern and central Europe (Italy was excluded from these analyses). We discuss in the next three paragraphs the likely reasons for this discrepancy.

Star-like shapes in sequence networks are commonly taken as evidence for population expansion, and this was also inferred for the aurochs considering the structure of the P clade [21,22]. The star-like expectation [48], however, assumes that all samples have the same age (isochronous sampling), which is not the case for the aurochs data. As recently shown by [38], the analysis of samples with different ages (heterochronous sampling) can introduce a systematic bias in some classical population genetics analyses, but the direction of the bias cannot be easily predicted. Using a simulation approach, we found that a signal of population expansion, as estimated by neutrality tests strictly dependent on the network shape, can be generated in a population with a constant size when individuals are sampled at the same time points as in the aurochs data set. In other words, the shape of the network in mtDNA sequences observed in northern and central Europe cannot be taken *per se *as evidence for population expansion.

More specifically, [22] concluded for the northern and central European aurochs that an exponential growth model gave a better fit than a constant size model, as indicated by marginal posterior probabilities. Using the same data set, and comparing the models not on the basis of the marginal posterior probabilities but on the basis of the marginal likelihoods using the more statistically sound Bayes factor, we reached different conclusions. There was no support for an exponential growth model over a constant size model. A similar result was obtained analysing the Italian aurochsen, where the Bayes factor in favour of a demographic change model, as compared to a constant population size model, was even smaller. The genetic data, therefore, do not support a demographic expansion and were more compatible with a model of constant population size. These results were confirmed when the Bayesian skyline plot was properly generated taking into account the temporal structure of the data.

The demographic expansion model for the aurochs was also suggested in a recent study on several bovine species [47]. However, in this analysis the estimated effective population size for the aurochs had a large, uninformative 95% credible interval of 3*10^4 ^- 9*10^9 ^and a median value 2*10^9 ^(after two independent MCMC with 10^8 ^steps using the input file provided in Ho et al. 2008). In addition, MCMC showed poor mixing and high autocorrelation for the population size and the growth rate. This multi-species approach is possibly uninformative regarding the aurochs demography, and requires further investigations (Simon Ho, personal communication).

The Pleistocene glacial and inter-glacial periods heavily impacted genetic variation in many species [6,8,10], and the steppe-tundra environment in central and northern Europe during the last glaciation was probably not suitable for the aurochs [5]. Does the genetic pattern we observed for the aurochs tell us something about the population dynamics in this species during the last severe climatic change in Europe?

First of all, genetic variation is higher in Italy compared to northern/central Europe (see also the effective population size estimates in Table 1), suggesting that a typical Southern refugium, Italy, might have acted as a variation reservoir during the glaciations. We can predict a certain degree of genetic homogeneity between Italy and other South-Eastern Mediterranean areas, considering that i) the Italian aurochs, and the European and Middle-Eastern cattle breeds, which likely descend mainly from aurochsen domesticated in the Fertile Crescent, share very similar mtDNA haplotypes; and ii) three early Neolithic specimens from Syria and Turkey, with a possible attribution to *Bos primigenius *[22,28], had T sequences, i.e. sequences belonging to the mtDNA clade typical in the Italian aurochs. It is therefore possible that aurochsen living in several Mediterranean regions, sharing similar ecological conditions and belonging to a species with a large migratory potential, exchanged enough genetic material to maintain a certain degree of homogeneity. Second, the low frequency of P sequences in Italy, which are the only sequences found in northern areas, indicates that these areas were not re-colonized by Italian or South-Eastern migrants. The high genetic divergence between Italian and northern/central European aurochsen is compatible with a long-term separation and small levels, if any, of gene flow. Third, the Bayesian analysis suggests that post-glacial warming and likely range expansions were not accompanied in the aurochs by a demographic expansion, at least an expansion intense enough to leave a clear signature on the pattern of genetic variation.

If these conclusions are correct, we should finally address the following question: where is the geographical origin of the aurochsen living after the last glaciation in the northern and central Europe, a region which was probably re-colonized in warmer times by a group of migrants without a significant demographic increase?

Armed with the current data we cannot rule out the hypothesis that the Iberian peninsula was a genetically divergent refugium in Southern Europe which contributed to the post-glacial colonization. We note however that the occurrence of continuous gene flow across southern Europe (including Iberia) was already proposed for the brown bear [14,49], supporting the idea that the model of southern refugia in Europe inhabited by genetically differentiated populations can be violated in some species. At the same time, the role of eastern, and not south-eastern, refugia, i.e. areas from the Carpathian to the Caspian regions, and further East in Asia, might have been important for the aurochs, as well as for other species [7,10,50,51]. We should not forget that some regions in Eastern Asia hosted temperate forests with large areas of grassland during the last glaciation [52], where relict populations of aurochsen could have survived before a post-glacial westwards migration. In this regard, it is intriguing to consider that a domestic individual with uncertain origin (possibly belonging to the Holstein breed) but with a P sequence was recently reported from a laboratory in Korea [23]. More recently, a survey of cytochrome b sequences in modern cattle found a P haplotype in another Holstein cattle as well as in an individual of the indigenous Korean Youngju Yellow breed [53]. Additional ancient DNA data from different areas will possibly clarify this issue.

### A final warning

Unfortunately, our conclusions regarding the northern and central European aurochsen, as well as those reported in all the other studies on the same topic (e.g.,[18,19,28]), rely on the assumption that T sequences were not present before domestication in that area. This is a plausible view, but the evidence supporting it is limited, since i) the number of pre-Neolithic specimens excavated and successfully typed from that area is small; ii) several individuals mainly from Germany, morphologically classified as "*Bos *sp", or "*Bos taurus*/*Bos primigenius*?", or "*Bos primigenius*?" or "*Bos taurus*?" had T haplotypes ([28], Supplementary Materials). It seems therefore that the idea that T haplotypes were absent in northern and central European aurochsen is not really based on the accumulation of data in favour of this hypothesis. Rather, this idea may have more to do with the results of early studies based on few aurochs sequences, and with some level of uncertainty in the morphological attribution of ancient skeletal remains. In the words of the authors, a more conservative conclusion should be "...there is no conclusive evidence of aurochs with a T haplotype in Europe" ([22], Supplementary Materials).

Of course, this conclusion should not be applied to Europe in general, since we have seen that a large fraction of Italian pre-Neolithic aurochsen (where the morphological attribution does not represent a problem) have T sequences. In conclusion, it is possible that the geographical distribution of T haplotypes before the arrival in Europe of domestic breeds was wider than commonly assumed. If this is true, the pattern of north-south population divergence in the aurochs could be less extreme than is described here, and the hypothesis that modern cattle breeds have ancestors not only in the Middle East and possibly in Italy[18], but also in other European regions [54], should be subjected to a robust investigation.

## Conclusion

The remarkable genetic difference between Italian aurochsen and the group of northern/central European aurochsen, and the similarity between the former and modern taurine breeds, is fully supported by the ancient mtDNA sequences typed in this study. Using extensive simulation and Bayesian based coalescent approaches we found no evidence of a demographic expansion in either Italian or northern/central European aurochsen groups. In fact, our observations lead us to conclude that the previously reported signatures may be artefactual. We interpret our results as evidence that the aurochs population sizes in southern Europe were not affected by climatic change in the last 30,000 years, and that central and northern areas in Europe were recolonized after the last glaciation by eastern immigrants without a major demographic expansion.

## Methods

### Samples collection and laboratory analyses

A total of 56 *Bos primigenius *samples from different well-studied sites across Italy were analyzed. To avoid the possible attributions of fragmented remains from domestic animals to *Bos primigenius*, we considered only samples dating before the Neolithic spread of domestication. Radiocarbon dating of associated remains (47 samples) or stratigraphic contests (for nine samples) were used to select the samples to analyse [55-57].

Details of each sample are provided in Table S1 (see Additional file 1). DNA was extracted for all samples in the Florence laboratory, and, for a subset of samples (see below), also in the Trento laboratory. Both laboratories are dedicated exclusively to ancient DNA work.

DNA extractions and PCR set up were carried out in rooms physically separated from those in which PCR cyclings and post-PCR analysis were conducted. Multiple measures were undertaken to exclude contamination and potential artifactual DNA changes (including post-mortem damage).

Disposable masks, gloves, and laboratory coats were worn during the experiments and were changed frequently. Pipettors were UV-irradiated in between use. DNA extractions and PCR reactions included negative controls. In each set of extractions or purifications, we included a negative control, represented by all the reagents except the bone powder, and these negative controls, together with blanks (all amplification reagents minus DNA), were regularly analysed in every PCR experiment to control for presence of exogenous DNA. All steps of the analysis were replicated at least twice. Seven bone specimens were independently extracted, amplified, cloned and sequenced in the ancient DNA laboratory of the Centre of Alpine Ecology (Edmund Mach Fundation, Trento). To test for preservation of other macromolecules as an indirect evidence for DNA survival [30,58] we estimated the degree of amino acid racemization in each sample. Approximately 5 mg of bone powder were used in this analysis [30]. The amount of target DNA was quantified in a sub-sample of specimens by Real Time (RT) PCR.

### Ancient DNA analysis in Florence

#### DNA extraction

All DNA-preparation and extraction methods followed strictly specific ancient DNA requirements [27,29,59]. To prevent contamination from prior handling, the outer layer of bones was removed with a rotary tool, and the fragments were briefly soaked in 10% bleach. All samples were then irradiated (1 hour under UV light) and powdered. DNA was extracted by means of a silica-based protocol (modified from [60]). Two independent extracts were obtained from each remain. Multiple negative controls were included in each extraction.

#### Quantification of DNA Molecules

Real-time PCR amplification was performed using Brilliant^® ^SYBR^® ^Green QPCR Master Mix (Stratagene) in MX3000P (Stratagene), using 0.5 μM of appropriate primers (reverse primer located at L16030 and forward primer located at H16165). Thermal cycling conditions were 95°C for 10 min, 40 cycles at 95°C for 30 s, 53°C for 1 min and 72°C for 30 s, followed by SYBR^® ^Green dissociation curve steep. Ten-fold serial dilutions of the purified and quantified standard were included in the experiment to create the standard curve in order to know the number of initial DNA molecules in the samples.

#### Amplification of mtDNA

Two μl of DNA extracted from the bone were amplified with this profile: 94°C for 10 min (Taq polymerase activation), followed by 50 cycles of PCR (denaturation, 94°C for 45 sec, annealing, 52°C for 1 min and extension, 72°C for 1 min) and final step at 72°C for 10 min. The 50 μl reaction mix contained 2 U of AmpliTaq Gold (Applied Biosystems), 200 μM of each dNTP and 1 μM of each primer. The HVR-I (Hyper variable region one of D-loop Mitochondrial DNA) was subdivided in three overlapping fragments of 134, 116, and 104 bais pairs, respectively. Primers used were: L16030 5'-ACATTAAATTATATGCCCCATGC-3'; H16165 5'-TTCACGCGGCATGGTA-3'; L16159 5'-'TTCCTTACCATTAGATCACGAGC-3; H16276 5' GATGAGATGGCCCTGAAGAA-3'; L16267 5'-CAATGAATTTTACCAGGCAT-3'; H00034 5'-CCAAATGTGACAGCACAG-3'. Each extract was amplified at least twice. Since overlapping primers were used throughout the PCR amplifications, it is highly unlikely that we amplified a nuclear insertion rather than the organellar mtDNA.

#### Cloning and Sequencing

PCR products were cloned using the TOPO TA Cloning Kit (Invitrogen) according to the manufacturer's instructions. Screening of white recombinant colonies was accomplished by PCR, transferring the colonies into a 30 μl reaction mix (67 mM Tris HCl [pH 8.8], 2 mM MgCl_2_, 1 μM of each primer, 0.125 mM of each dNTP, 0.75 units of Taq Polymerase) containing M13 forward and reverse universal primers. After 5 min at 92°C, 30 cycles of PCR (30 sec at 90°C, 1 min at 50°C, 1 min at 72°C) were carried out and clones with insert of the expected size were identified by agarose gel electrophoresis. After purification of these PCR products with Microcon PCR devices (Amicon), a volume of 1,5 μl was cycle-sequenced following the BigDye Terminator kit (Applied Biosystems) using the supplier's instructions. The sequence was determined using an Applied BioSystems 3100 DNA sequencer. The clones sequences for each sample are reported in Table S2 (see Additional file 2).

### Independent replications in Trento

The first DNA fragment, which includes most of the variable sites, was replicated for seven bone samples in the CEA laboratory in Trento. The same ancient DNA protocols followed in Florence were adopted in Trento, with the exception of the Strata Clone TM PCR Cloning Kit.

### Statistical analyses

A median-joining network [61] among aurochs haplotypes, defined by the segregating sites of the partial control region stretch (120 bp), was constructed using the software NETWORK 4.5.0.0 (available at http://fluxus-engineering.com) with default settings. The level of divergence between populations/breeds was estimated using the analysis of molecular variance [62], as implemented in ARLEQUIN 3.11 [63]. Pairwise Φ*st *values were simply computed using the number of differences as a molecular distance between alleles, with standard errors based on 1,000 bootstrap replicates. The distance matrix was plotted in two dimensions by means of multidimensional scaling (MDS) using the ALSCAL algorithm implemented in the SPSS 12.0 software package.

The demography of the Italian aurochs population was investigated with the software BEAST 1.4.7 [64] using two different coalescence prior models (constant size and exponential change) and the HKY model of nucleotide substitution, as selected by MODELTEST 3.7 according to the hierarchical likelihood ratio test method [65]. The transition/transversion ratio could not be estimated from the data (because of the presence of a single transversion in one sample only) and was set as in [22]; final results are based on Ti/Tv = 50. The demographic models were run three times for 10,000,000 MCMC iterations with a 20% burnin and a thinning interval of 1,000, and were compared by means of the Bayes Factor. Bayes Factor was computed as twice the difference between the log of the marginal likelihoods, which were approximated using the harmonic mean as suggested by [34]. Convergence was checked by examining the generation plot visualized with TRACER 1.3 [66] and the final estimates were based on the pooled runs (after removing the burnin). TMRCA of the whole sample as well as of the T and P haplogroups were recorded within both demographic models, after checking that T and P were monophyletic with posterior probabilities of 1.00 (hence confirming that the partial control region contained enough information to distinguish between the two haplogroups). The mutation rate was estimated from the data, using the ages of the sequences to calibrate the clock. This procedure is considered more appropriate than using an external calibration point [67].

To check if the demography of the Italian aurochs differed from that estimated for the northern and central European aurochs, we analysed the data in [22] with the same settings as above. We also explored the population history of the European aurochs to determine whether heterochronous sampling could skew the distribution of summary statistics used to detect changes in the effective population size. In this case, we performed a grid of simulation with various combinations of *Ne *and growth rates under a temporal sampling scheme analogous to that in [22]. Generation time was set to seven years [35] and mutation rate to 7.6 × 10^-7 ^per site per year (mean of the posterior distribution of the mutation rate estimated by [22]). The software SERIALSIMCOAL [68] was used for the simulations. For each scenario we performed 1,000 replicates, and Tajima's *D *and Fu' *Fs *were computed with ARLEQUIN 3.11 [63] as indices of population size changes. The empirical distribution of these statistics was reconstructed under various models, and compared to the null distributions expected assuming an isochronous sampling scheme under the same coalescent models. For each simulated scenario we computed the percentage of significant tests using the procedure implemented in ARLEQUIN (i.e., assuming an isochronous sampling scheme).

Finally, a Bayesian skyline plot [69] was constructed for both the Italian and the northern/central European aurochs population. We ran the analysis twice for each group. Length of the MCMC was set to 20,000,000 iterations with a 10% burnin and a thinning interval of 1,000. The gene genealogy was divided into three internodes groups and effective population size function was fitted with a piecewise constant function of population size change. The reconstructed skyline was also compared for both groups with the skyline estimated assuming an isochronous sampling scheme.

## Abbreviations

MYA: Millions of years; MCMC: Monte Carlo Markov Chain; TMRCA: Time to the Most Recent Common Ancestor; BF: Bayes Factor.

## Authors' contributions

GC, ML, EP and GL performed aDNA laboratory analyses. AC performed the racemization analysis. PB, LS and CDP provided samples and radiocarbon/stratigraphic information. SM and GB performed the statistical analyses. GB and DC conceived the project. GB, DC, SM and GC wrote the paper. All authors read and approved the manuscript.

## Supplementary Material

Additional file 1 **Table S1**. Summary of the major characteristics of the samples, including radiocarbon dates. Click here for file

Additional file 2 **Table S2**. The clone sequences. Click here for file

Additional file 3 **Table S3**. The consensus sequence for each sample. Click here for file

Additional file 4 **Table S4**. The geographic origin, name, samples size, and reference for all the taurine breeds considered in this study. Click here for file
