# Supplementary material for: Population dynamic of the extinct European aurochs: genetic evidence of a north-south differentiation pattern and no evidence of post-glacial expansion
Source: BMC Evol Biol. 2010 Mar 26;10:83. doi: 10.1186/1471-2148-10-83 (PMC2858146; doi:10.1186/1471-2148-10-83)
Supplement: Additional file 2 — Table S2. The clone sequences. [file 1471-2148-10-83-S2.DOC]

Table S2. Clone sequences. The first line reports the reference sequence (BRS) with the numbering of the nucleotide positions. Nucleotides identical to the reference sequence are indicated by dots. Clones are identified by an abbreviation and two numbers: the abbreviation refers to the laboratory (FLO: Florence; CEA: Centre of Alpine Ecology, Trento); the first number indicates the extraction; the second number indicates the PCR.

11111111111111111111111111111111111111111111111111111111111111111111111111111111111111111111111111111111111111111111111111111111111111111111111111111111111111111111111111111111111111111111111111111111111111111111111111111111111111111111111111111111111111111111111111111111111111111111111111111111111111111111000000000000000000000000000000000

66666666666666666666666666666666666666666666666666666666666666666666666666666666666666666666666666666666666666666666666666666666666666666666666666666666666666666666666666666666666666666666666666666666666666666666666666666666666666666666666666666666666666666666666666666666666666666666666666666666666666666666000000000000000000000000000000000

00000000000000000000000000000000000000000000000000000000000000000000011111111111111111111111111111111111111111111111111111111111111111111111111111111111111111111111111112222222222222222222222222222222222222222222222222222222222222222222222222222222222222222222222222222333333333333333333333333333333333333333000000000000000000000000000000000

33333333344444444445555555555666666666677777777778888888888999999999900000000001111111111222222222233333333334444444444555555555566666666667777777777888888888899999999990000000000111111111122222222223333333333444444444455555555556666666666777777777788888888889999999999000000000011111111112222222222333333333000000000111111111122222222223333

12345678901234567890123456789012345678901234567890123456789012345678901234567890123456789012345678901234567890123456789012345678901234567890123456789012345678901234567890123456789012345678901234567890123456789012345678901234567890123456789012345678901234567890123456789012345678901234567890123456789012345678123456789012345678901234567890123

REF. ATATAAGCAAGTACATGACCTCTATAGCAGTACATAATACATATAATTATTGACTGTACATAGTACATTATGTCAAATTCATTCTTGATAGTATATCTATTATATATTCCTTACCATTAGATCACGAGCTTAATTACCATGCCGCGTGAAACCAGCAACCCGCTAGGCAGGGATCCCTCTTCTCGCTCCGGGCCCATAAACCGTGGGGGTCGCTATCCAATGAATTTTACCAGGCATCTGGTTCTTTCTTCAGGGCCATCTCATCTAAAACGGTCCATTCTTTCCTCTTAAATAAGACATCTCGATGGACTAATGGCTAATCAGCCCATGCTCACACATAA

Au-It6

FLO 1.1 ......................................................................................................................................TACCATGCCGCGTGAA

FLO 1.1 ......................................................................................................................................

FLO 1.2 ......................................................................................................................................

FLO 1.2 ......................................................................................................................................

FLO 2.1 ......................................................................................................................................

FLO 2.1 ......................................................................................................................................

FLO 2.2 ......................................................................................................................................

FLO 2.2 ......................................................................................................................................

CEA 1.1 ......................................................................................................................................

CEA 1.1 ..........................T...........................................................................................................

CEA 1.1 ........................................................................T.............................................................

CEA 1.1 ......................................................................................................................................

CEA 1.1 ......................................................................................................................................

FLO 1.1 TTCCTTACCATTAGATCACGAGC....................................................................................................................TTCTTCAGGGCCATCTCATC

FLO 1.1 ....................................................................................................................

FLO 1.2 ....................................................................................................................

FLO 1.2 ....................................................................................................................

FLO 2.1 ....................................................................................................................

FLO 2.1 ....................................................................................................................

FLO 2.2 ....................................................................................................................

FLO 2.2 ....................................................................................................................

Consensus ATATAAGCAAGTACATGACCTCTATAGCAGTACATAATACATATAATTATTGACTGTACATAGTACATTATGTCAAATTCATTCTTGATAGTATATCTATTATATATTCCTTACCATTAGATCACGAGCTTAATTACCATGCCGCGTGAAACCAGCAACCCGCTAGGCAGGGATCCCTCTTCTCGCTCCGGGCCCATAAACCGTGGGGGTCGCTATCCAATGAATTTTACCAGGCATCTGGTTCT

11111111111111111111111111111111111111111111111111111111111111111111111111111111111111111111111111111111111111111111111111111111111111111111111111111111111111111111111111111111111111111111111111111111111111111111111111111111111111111111111111111111111111111111111111111111111111111111111111111111111111111111000000000000000000000000000000000

66666666666666666666666666666666666666666666666666666666666666666666666666666666666666666666666666666666666666666666666666666666666666666666666666666666666666666666666666666666666666666666666666666666666666666666666666666666666666666666666666666666666666666666666666666666666666666666666666666666666666666666000000000000000000000000000000000

00000000000000000000000000000000000000000000000000000000000000000000011111111111111111111111111111111111111111111111111111111111111111111111111111111111111111111111111112222222222222222222222222222222222222222222222222222222222222222222222222222222222222222222222222222333333333333333333333333333333333333333000000000000000000000000000000000

33333333344444444445555555555666666666677777777778888888888999999999900000000001111111111222222222233333333334444444444555555555566666666667777777777888888888899999999990000000000111111111122222222223333333333444444444455555555556666666666777777777788888888889999999999000000000011111111112222222222333333333000000000111111111122222222223333

12345678901234567890123456789012345678901234567890123456789012345678901234567890123456789012345678901234567890123456789012345678901234567890123456789012345678901234567890123456789012345678901234567890123456789012345678901234567890123456789012345678901234567890123456789012345678901234567890123456789012345678123456789012345678901234567890123

REF. ATATAAGCAAGTACATGACCTCTATAGCAGTACATAATACATATAATTATTGACTGTACATAGTACATTATGTCAAATTCATTCTTGATAGTATATCTATTATATATTCCTTACCATTAGATCACGAGCTTAATTACCATGCCGCGTGAAACCAGCAACCCGCTAGGCAGGGATCCCTCTTCTCGCTCCGGGCCCATAAACCGTGGGGGTCGCTATCCAATGAATTTTACCAGGCATCTGGTTCTTTCTTCAGGGCCATCTCATCTAAAACGGTCCATTCTTTCCTCTTAAATAAGACATCTCGATGGACTAATGGCTAATCAGCCCATGCTCACACATAA

Au-It7

FLO 1.1 .......................................................................................CG.............................................TACCATGCCGCGTGAA

FLO 1.1 ......................................................................................................................................

FLO 1.2 ......................................................................................................................................

FLO 1.2 ......................................................................................................................................

FLO 2.1 ......................................................................................................................................

FLO 2.1 ......................................................................................................................................

FLO 2.2 ......................................................................................................................................

FLO 2.2 ......................................................................................................................................

CEA 1.1 ......................................................................................................................................

CEA 1.1 ......................................................................................................................................

CEA 1.1 .................................................................T....................................................................

CEA 1.1 ......................................................................................A...............................................

CEA 1.1 ......................................................................................................................................

Consensus ATATAAGCAAGTACATGACCTCTATAGCAGTACATAATACATATAATTATTGACTGTACATAGTACATTATGTCAAATTCATTCTTGATAGTATATCTATTATATATTCCTTACCATTAGATCACGAGCTTAAT

11111111111111111111111111111111111111111111111111111111111111111111111111111111111111111111111111111111111111111111111111111111111111111111111111111111111111111111111111111111111111111111111111111111111111111111111111111111111111111111111111111111111111111111111111111111111111111111111111111111111111111111000000000000000000000000000000000

66666666666666666666666666666666666666666666666666666666666666666666666666666666666666666666666666666666666666666666666666666666666666666666666666666666666666666666666666666666666666666666666666666666666666666666666666666666666666666666666666666666666666666666666666666666666666666666666666666666666666666666000000000000000000000000000000000

00000000000000000000000000000000000000000000000000000000000000000000011111111111111111111111111111111111111111111111111111111111111111111111111111111111111111111111111112222222222222222222222222222222222222222222222222222222222222222222222222222222222222222222222222222333333333333333333333333333333333333333000000000000000000000000000000000

33333333344444444445555555555666666666677777777778888888888999999999900000000001111111111222222222233333333334444444444555555555566666666667777777777888888888899999999990000000000111111111122222222223333333333444444444455555555556666666666777777777788888888889999999999000000000011111111112222222222333333333000000000111111111122222222223333

12345678901234567890123456789012345678901234567890123456789012345678901234567890123456789012345678901234567890123456789012345678901234567890123456789012345678901234567890123456789012345678901234567890123456789012345678901234567890123456789012345678901234567890123456789012345678901234567890123456789012345678123456789012345678901234567890123

REF. ATATAAGCAAGTACATGACCTCTATAGCAGTACATAATACATATAATTATTGACTGTACATAGTACATTATGTCAAATTCATTCTTGATAGTATATCTATTATATATTCCTTACCATTAGATCACGAGCTTAATTACCATGCCGCGTGAAACCAGCAACCCGCTAGGCAGGGATCCCTCTTCTCGCTCCGGGCCCATAAACCGTGGGGGTCGCTATCCAATGAATTTTACCAGGCATCTGGTTCTTTCTTCAGGGCCATCTCATCTAAAACGGTCCATTCTTTCCTCTTAAATAAGACATCTCGATGGACTAATGGCTAATCAGCCCATGCTCACACATAA

Au-It8

FLO 1.1 ......................................................................................................................................TACCATGCCGCGTGAA

FLO 1.1 ......................................................................................................................................

FLO 1.2 ......................................................................................................................................

FLO 1.2 ......................................................................................................................................

FLO 1.2 ......................................................................................................................................

FLO 2.1 ......................................................................................................................................

FLO 2.1 ......................................................................................................................................

FLO 2.2 ......................................................................................................................................

FLO 2.2 ......................................................................................................................................

CEA 1.1 .....................................................T................................................................................

CEA 1.1 ...........A..........................................................................................................................

CEA 1.1 .......................................................A..............................................................................

CEA 1.1 .......................................................A..............................................................................

CEA 1.1 .............................A........................................................................................................

FLO 1.1 CAATGAATTTTACCAGGCAT........................................................................................................

FLO 1.1 ........................................................................................................

FLO 1.2 ........................................................................................................

FLO 1.2 ........................................................................................................

FLO 1.2 ........................................................................................................

FLO 2.1 ........................................................................................................

FLO 2.1 ........................................................................................................

FLO 2.2 ......................................A.................................................................

FLO 2.2 ........................................................................................................

Consensus ATATAAGCAAGTACATGACCTCTATAGCAGTACATAATACATATAATTATTGACTGTACATAGTACATTATGTCAAATTCATTCTTGATAGTATATCTATTATATATTCCTTACCATTAGATCACGAGCTTAAT CTGGTTCTTTCTTCAGGGCCATCTCATCTAAAACGGTCCATTCTTTCCTCTTAAATAAGACATCTCGATGGACTAATGGCTAATCAGCCCATGCTCACACATAA

11111111111111111111111111111111111111111111111111111111111111111111111111111111111111111111111111111111111111111111111111111111111111111111111111111111111111111111111111111111111111111111111111111111111111111111111111111111111111111111111111111111111111111111111111111111111111111111111111111111111111111111000000000000000000000000000000000

66666666666666666666666666666666666666666666666666666666666666666666666666666666666666666666666666666666666666666666666666666666666666666666666666666666666666666666666666666666666666666666666666666666666666666666666666666666666666666666666666666666666666666666666666666666666666666666666666666666666666666666000000000000000000000000000000000

00000000000000000000000000000000000000000000000000000000000000000000011111111111111111111111111111111111111111111111111111111111111111111111111111111111111111111111111112222222222222222222222222222222222222222222222222222222222222222222222222222222222222222222222222222333333333333333333333333333333333333333000000000000000000000000000000000

33333333344444444445555555555666666666677777777778888888888999999999900000000001111111111222222222233333333334444444444555555555566666666667777777777888888888899999999990000000000111111111122222222223333333333444444444455555555556666666666777777777788888888889999999999000000000011111111112222222222333333333000000000111111111122222222223333

12345678901234567890123456789012345678901234567890123456789012345678901234567890123456789012345678901234567890123456789012345678901234567890123456789012345678901234567890123456789012345678901234567890123456789012345678901234567890123456789012345678901234567890123456789012345678901234567890123456789012345678123456789012345678901234567890123

REF. ATATAAGCAAGTACATGACCTCTATAGCAGTACATAATACATATAATTATTGACTGTACATAGTACATTATGTCAAATTCATTCTTGATAGTATATCTATTATATATTCCTTACCATTAGATCACGAGCTTAATTACCATGCCGCGTGAAACCAGCAACCCGCTAGGCAGGGATCCCTCTTCTCGCTCCGGGCCCATAAACCGTGGGGGTCGCTATCCAATGAATTTTACCAGGCATCTGGTTCTTTCTTCAGGGCCATCTCATCTAAAACGGTCCATTCTTTCCTCTTAAATAAGACATCTCGATGGACTAATGGCTAATCAGCCCATGCTCACACATAA

Au-It9

FLO 1.1 ......................................................................................................................................TACCATGCCGCGTGAA

FLO 1.1 ......................................................................................................................................

FLO 1.2 ......................................................................................................................................

FLO 1.2 ......................................................................................................................................

FLO 2.1 ......................................................................................................................................

FLO 2.1 ......................................................................................................................................

FLO 2.2 ......................................................................................................................................

FLO 2.2 ......................................................................................................................................

Consensus ATATAAGCAAGTACATGACCTCTATAGCAGTACATAATACATATAATTATTGACTGTACATAGTACATTATGTCAAATTCATTCTTGATAGTATATCTATTATATATTCCTTACCATTAGATCACGAGCTTAAT

11111111111111111111111111111111111111111111111111111111111111111111111111111111111111111111111111111111111111111111111111111111111111111111111111111111111111111111111111111111111111111111111111111111111111111111111111111111111111111111111111111111111111111111111111111111111111111111111111111111111111111111000000000000000000000000000000000

66666666666666666666666666666666666666666666666666666666666666666666666666666666666666666666666666666666666666666666666666666666666666666666666666666666666666666666666666666666666666666666666666666666666666666666666666666666666666666666666666666666666666666666666666666666666666666666666666666666666666666666000000000000000000000000000000000

00000000000000000000000000000000000000000000000000000000000000000000011111111111111111111111111111111111111111111111111111111111111111111111111111111111111111111111111112222222222222222222222222222222222222222222222222222222222222222222222222222222222222222222222222222333333333333333333333333333333333333333000000000000000000000000000000000

33333333344444444445555555555666666666677777777778888888888999999999900000000001111111111222222222233333333334444444444555555555566666666667777777777888888888899999999990000000000111111111122222222223333333333444444444455555555556666666666777777777788888888889999999999000000000011111111112222222222333333333000000000111111111122222222223333

12345678901234567890123456789012345678901234567890123456789012345678901234567890123456789012345678901234567890123456789012345678901234567890123456789012345678901234567890123456789012345678901234567890123456789012345678901234567890123456789012345678901234567890123456789012345678901234567890123456789012345678123456789012345678901234567890123

REF. ATATAAGCAAGTACATGACCTCTATAGCAGTACATAATACATATAATTATTGACTGTACATAGTACATTATGTCAAATTCATTCTTGATAGTATATCTATTATATATTCCTTACCATTAGATCACGAGCTTAATTACCATGCCGCGTGAAACCAGCAACCCGCTAGGCAGGGATCCCTCTTCTCGCTCCGGGCCCATAAACCGTGGGGGTCGCTATCCAATGAATTTTACCAGGCATCTGGTTCTTTCTTCAGGGCCATCTCATCTAAAACGGTCCATTCTTTCCTCTTAAATAAGACATCTCGATGGACTAATGGCTAATCAGCCCATGCTCACACATAA

Au-It10

FLO 1.1 ..........................CT..........................................................................................................TACCATGCCGCGTGAA

FLO 1.1 ..........................CT..........................................................................................................

FLO 1.2 ..........................CT.........................G................................................................................

FLO 1.2 ..........................CT..........................................................................................................

FLO 2.1 ..........................CT..........................................................................................................

FLO 2.1 ..........................CT..........................................................................................................

FLO 2.2 ..........................CT..........................................................................................................

FLO 2.2 ..........................CT..........................................................................................................

Consensus ATATAAGCAAGTACATGACCTCTATACTAGTACATAATACATATAATTATTGACTGTACATAGTACATTATGTCAAATTCATTCTTGATAGTATATCTATTATATATTCCTTACCATTAGATCACGAGCTTAAT

11111111111111111111111111111111111111111111111111111111111111111111111111111111111111111111111111111111111111111111111111111111111111111111111111111111111111111111111111111111111111111111111111111111111111111111111111111111111111111111111111111111111111111111111111111111111111111111111111111111111111111111000000000000000000000000000000000

66666666666666666666666666666666666666666666666666666666666666666666666666666666666666666666666666666666666666666666666666666666666666666666666666666666666666666666666666666666666666666666666666666666666666666666666666666666666666666666666666666666666666666666666666666666666666666666666666666666666666666666000000000000000000000000000000000

00000000000000000000000000000000000000000000000000000000000000000000011111111111111111111111111111111111111111111111111111111111111111111111111111111111111111111111111112222222222222222222222222222222222222222222222222222222222222222222222222222222222222222222222222222333333333333333333333333333333333333333000000000000000000000000000000000

33333333344444444445555555555666666666677777777778888888888999999999900000000001111111111222222222233333333334444444444555555555566666666667777777777888888888899999999990000000000111111111122222222223333333333444444444455555555556666666666777777777788888888889999999999000000000011111111112222222222333333333000000000111111111122222222223333

12345678901234567890123456789012345678901234567890123456789012345678901234567890123456789012345678901234567890123456789012345678901234567890123456789012345678901234567890123456789012345678901234567890123456789012345678901234567890123456789012345678901234567890123456789012345678901234567890123456789012345678123456789012345678901234567890123

REF. ATATAAGCAAGTACATGACCTCTATAGCAGTACATAATACATATAATTATTGACTGTACATAGTACATTATGTCAAATTCATTCTTGATAGTATATCTATTATATATTCCTTACCATTAGATCACGAGCTTAATTACCATGCCGCGTGAAACCAGCAACCCGCTAGGCAGGGATCCCTCTTCTCGCTCCGGGCCCATAAACCGTGGGGGTCGCTATCCAATGAATTTTACCAGGCATCTGGTTCTTTCTTCAGGGCCATCTCATCTAAAACGGTCCATTCTTTCCTCTTAAATAAGACATCTCGATGGACTAATGGCTAATCAGCCCATGCTCACACATAA

Au-It11

FLO 1.1 ............................................................................................... T.....................................TACCATGCCGCGTGAA

FLO 1.1 ..........................................................................C.....................T.....................................

FLO 1.2 ................................................................................................T.....................................

FLO 1.2 ................................................................................................T.....................................

FLO 2.1 .......................................................................................G........T.....................................

FLO 2.1 ................................................................................................T.....................................

FLO 2.2 ................................................................................................T.....................................

FLO 2.2 ................................................................................................T.....................................

Consensus ATATAAGCAAGTACATGACCTCTATAGCAGTACATAATACATATAATTATTGACTGTACATAGTACATTATGTCAAATTCATTCTTGATAGTATATTTATTATATATTCCTTACCATTAGATCACGAGCTTAAT

11111111111111111111111111111111111111111111111111111111111111111111111111111111111111111111111111111111111111111111111111111111111111111111111111111111111111111111111111111111111111111111111111111111111111111111111111111111111111111111111111111111111111111111111111111111111111111111111111111111111111111111000000000000000000000000000000000

66666666666666666666666666666666666666666666666666666666666666666666666666666666666666666666666666666666666666666666666666666666666666666666666666666666666666666666666666666666666666666666666666666666666666666666666666666666666666666666666666666666666666666666666666666666666666666666666666666666666666666666000000000000000000000000000000000

00000000000000000000000000000000000000000000000000000000000000000000011111111111111111111111111111111111111111111111111111111111111111111111111111111111111111111111111112222222222222222222222222222222222222222222222222222222222222222222222222222222222222222222222222222333333333333333333333333333333333333333000000000000000000000000000000000

33333333344444444445555555555666666666677777777778888888888999999999900000000001111111111222222222233333333334444444444555555555566666666667777777777888888888899999999990000000000111111111122222222223333333333444444444455555555556666666666777777777788888888889999999999000000000011111111112222222222333333333000000000111111111122222222223333

12345678901234567890123456789012345678901234567890123456789012345678901234567890123456789012345678901234567890123456789012345678901234567890123456789012345678901234567890123456789012345678901234567890123456789012345678901234567890123456789012345678901234567890123456789012345678901234567890123456789012345678123456789012345678901234567890123

REF. ATATAAGCAAGTACATGACCTCTATAGCAGTACATAATACATATAATTATTGACTGTACATAGTACATTATGTCAAATTCATTCTTGATAGTATATCTATTATATATTCCTTACCATTAGATCACGAGCTTAATTACCATGCCGCGTGAAACCAGCAACCCGCTAGGCAGGGATCCCTCTTCTCGCTCCGGGCCCATAAACCGTGGGGGTCGCTATCCAATGAATTTTACCAGGCATCTGGTTCTTTCTTCAGGGCCATCTCATCTAAAACGGTCCATTCTTTCCTCTTAAATAAGACATCTCGATGGACTAATGGCTAATCAGCCCATGCTCACACATAA

Au-It12

FLO 1.1 ......................................................................................................................................TACCATGCCGCGTGAA

FLO 1.1 ......................................................................................................................................

FLO 1.1 ......................................................................................................................................

FLO 1.2 ......................................................................................................................................

FLO 1.2 ......................C.................................................................C.............................................

FLO 2.1 ......................................................................................................................................

FLO 2.1 ......................................................................................................................................

FLO 2.1 ......................................................................................................................................

FLO 2.2 ......................C...............................................................................................................

FLO 2.2 ......................C...............................................................................................................

CEA 1.1 ......................................................................................................................................

CEA 1.1 ......................................................................................................................................

CEA 1.1 ...................................................A..................................................................................

CEA 1.1 ......................................................................................................................................

CEA 1.1 ......................................................................................................................................

FLO 1.1 TTCCTTACCATTAGATCACGAGC....................................................................................................................TTCTTCAGGGCCATCTCATC

FLO 1.1 ....................................................................................................................

FLO 1.1 ....................................................................................................................

FLO 1.2 ....................................................................................................................

FLO 1.2 ....................................................................................................................

FLO 2.1 ....................................................................................................................

FLO 2.1 ....................................................................................................................

FLO 2.2 ....................................................................................................................

FLO 2.2 ....................................................................................................................

FLO 2.2 ....................................................................................................................

FLO 1.1 CAATGAATTTTACCAGGCAT........................................................................................................

FLO 1.1 ........................................................................................................

FLO 1.1 ........................................................................................................

FLO 1.2 .........................................C..............................................................

FLO 1.2 ........................................................................................................

FLO 2.1 .........................................C..............................................................

FLO 2.1 ........................................................................................................

FLO 2.2 .........................................C..............................................................

FLO 2.2 ........................................................................................................

Consensus ATATAAGCAAGTACATGACCTCTATAGCAGTACATAATACATATAATTATTGACTGTACATAGTACATTATGTCAAATTCATTCTTGATAGTATATCTATTATATATTCCTTACCATTAGATCACGAGCTTAATTACCATGCCGCGTGAAACCAGCAACCCGCTAGGCAGGGATCCCTCTTCTCGCTCCGGGCCCATAAACCGTGGGGGTCGCTATCCAATGAATTTTACCAGGCATCTGGTTCTTTCTTCAGGGCCATCTCATCTAAAACGGTCCATTCTTTCCTCTTAAATAAGACATCTCGATGGACTAATGGCTAATCAGCCCATGCTCACACATAA

11111111111111111111111111111111111111111111111111111111111111111111111111111111111111111111111111111111111111111111111111111111111111111111111111111111111111111111111111111111111111111111111111111111111111111111111111111111111111111111111111111111111111111111111111111111111111111111111111111111111111111111000000000000000000000000000000000

66666666666666666666666666666666666666666666666666666666666666666666666666666666666666666666666666666666666666666666666666666666666666666666666666666666666666666666666666666666666666666666666666666666666666666666666666666666666666666666666666666666666666666666666666666666666666666666666666666666666666666666000000000000000000000000000000000

00000000000000000000000000000000000000000000000000000000000000000000011111111111111111111111111111111111111111111111111111111111111111111111111111111111111111111111111112222222222222222222222222222222222222222222222222222222222222222222222222222222222222222222222222222333333333333333333333333333333333333333000000000000000000000000000000000

33333333344444444445555555555666666666677777777778888888888999999999900000000001111111111222222222233333333334444444444555555555566666666667777777777888888888899999999990000000000111111111122222222223333333333444444444455555555556666666666777777777788888888889999999999000000000011111111112222222222333333333000000000111111111122222222223333

12345678901234567890123456789012345678901234567890123456789012345678901234567890123456789012345678901234567890123456789012345678901234567890123456789012345678901234567890123456789012345678901234567890123456789012345678901234567890123456789012345678901234567890123456789012345678901234567890123456789012345678123456789012345678901234567890123

REF. ATATAAGCAAGTACATGACCTCTATAGCAGTACATAATACATATAATTATTGACTGTACATAGTACATTATGTCAAATTCATTCTTGATAGTATATCTATTATATATTCCTTACCATTAGATCACGAGCTTAATTACCATGCCGCGTGAAACCAGCAACCCGCTAGGCAGGGATCCCTCTTCTCGCTCCGGGCCCATAAACCGTGGGGGTCGCTATCCAATGAATTTTACCAGGCATCTGGTTCTTTCTTCAGGGCCATCTCATCTAAAACGGTCCATTCTTTCCTCTTAAATAAGACATCTCGATGGACTAATGGCTAATCAGCCCATGCTCACACATAA

Au-It13

FLO 1.1 ..........A.......T.C......T...............C..........C....................................C..........................................TACCATGCCGCGTGAA

FLO 1.1 ..........A.......T.C......T...............C..........C....................................C..........................................

FLO 1.2 ..........A.......T.C......T...............C..........C....................................C..........................................

FLO 1.2 ..........A.......T.C......T...............C..........C....................................C..........................................

FLO 2.1 ..........A.......T.C......T...............C..........C....................................C..........................................

FLO 2.1 ..........A.......T.C......T...............C..........C....................................C..........................................

FLO 2.2 ..........A.......T.C......T...............C..........C....................................C..........................................

FLO 2.2 ..........A.......T.C......T...............C..........C....................................C..........................................

CEA 1.1 ..........A.......T.C......T...............C..........C....................................C..........................................

CEA 1.1 ..........A.......T.C......T...............C..........C....................................C..........................................

CEA 1.1 ..........A.......T.C......T...............C..........C....................................C..........................................

CEA 1.1 ..........A.......T.C......T...............C..........C....................................C..........................................

CEA 1.1 ..........A.......T.C......T...............C..........C....................................C..........................................

Consensus ATATAAGCAAATACATGATCCCTATAGTAGTACATAATACATACAATTATTGACCGTACATAGTACATTATGTCAAATTCATTCTTGATAGCATATCTATTATATATTCCTTACCATTAGATCACGAGCTTAAT

11111111111111111111111111111111111111111111111111111111111111111111111111111111111111111111111111111111111111111111111111111111111111111111111111111111111111111111111111111111111111111111111111111111111111111111111111111111111111111111111111111111111111111111111111111111111111111111111111111111111111111111000000000000000000000000000000000

66666666666666666666666666666666666666666666666666666666666666666666666666666666666666666666666666666666666666666666666666666666666666666666666666666666666666666666666666666666666666666666666666666666666666666666666666666666666666666666666666666666666666666666666666666666666666666666666666666666666666666666000000000000000000000000000000000

00000000000000000000000000000000000000000000000000000000000000000000011111111111111111111111111111111111111111111111111111111111111111111111111111111111111111111111111112222222222222222222222222222222222222222222222222222222222222222222222222222222222222222222222222222333333333333333333333333333333333333333000000000000000000000000000000000

33333333344444444445555555555666666666677777777778888888888999999999900000000001111111111222222222233333333334444444444555555555566666666667777777777888888888899999999990000000000111111111122222222223333333333444444444455555555556666666666777777777788888888889999999999000000000011111111112222222222333333333000000000111111111122222222223333

12345678901234567890123456789012345678901234567890123456789012345678901234567890123456789012345678901234567890123456789012345678901234567890123456789012345678901234567890123456789012345678901234567890123456789012345678901234567890123456789012345678901234567890123456789012345678901234567890123456789012345678123456789012345678901234567890123

REF. ATATAAGCAAGTACATGACCTCTATAGCAGTACATAATACATATAATTATTGACTGTACATAGTACATTATGTCAAATTCATTCTTGATAGTATATCTATTATATATTCCTTACCATTAGATCACGAGCTTAATTACCATGCCGCGTGAAACCAGCAACCCGCTAGGCAGGGATCCCTCTTCTCGCTCCGGGCCCATAAACCGTGGGGGTCGCTATCCAATGAATTTTACCAGGCATCTGGTTCTTTCTTCAGGGCCATCTCATCTAAAACGGTCCATTCTTTCCTCTTAAATAAGACATCTCGATGGACTAATGGCTAATCAGCCCATGCTCACACATAA

Au-It14

FLO 1.1 ..................T.C......T...............C..........C....................................C..........................................TACCATGCCGCGTGAA

FLO 1.1 ..................T.C......T...............C..........C....................................C..........................................

FLO 1.2 ..................T.C......T...............C..........C....................................C..........................................

FLO 1.2 ..................T.C......T...............C..........C....................................C..........................................

FLO 2.1 ..................T.C......T...............C..........C....................................C..........................................

FLO 2.1 ..................T.C......T...............C..........C....................................C..........................................

FLO 2.2 ..................T.C......T...............C..........C....................................C..........................................

FLO 2.2 ..................T.C......T...............C..........C....................................C..........................................

CEA 1.1 ..................T.C......T...............C..........C....................................C..........................................

CEA 1.1 ..................T.C......T....T..........C..........C....................................C..........................................

CEA 1.1 ..................T.C......T...............C..........C....................................C..........................................

CEA 1.1 ..................T.C......T...............C..........C....................................C..........................................

CEA 1.1 ..................T.C......T.A.............C..........C....................................C..........................................

FLO 1.1 TTCCTTACCATTAGATCACGAGC.................................T......A......T.......................T............................................TTCTTCAGGGCCATCTCATC

FLO 1.1 .................................T......A......T.......................T............................................

FLO 1.2 .................................T......A......T.......................T............................................

FLO 1.2 .................................T......A......T.......................T............................................

FLO 2.1 .................................T......A......T.......................T............................................

FLO 2.1 .................................T......A......T.......................T............................................

FLO 2.2 .................................T......A......T.......................T............................................

FLO 2.2 .................................T......A......T.......................T............................................

Consensus ATATAAGCAAGTACATGATCCCTATAGTAGTACATAATACATACAATTATTGACCGTACATAGTACATTATGTCAAATTCATTCTTGATAGCATATCTATTATATATTCCTTACCATTAGATCACGAGCTTAATTACCATGCCGCGTGAAACCAGCAACCCGTTAGGCAAGGATCCTTCTTCTCGCTCCGGGCCCATAAATCGTGGGGGTCGCTATCCAATGAATTTTACCAGGCATCTGGTTCT

11111111111111111111111111111111111111111111111111111111111111111111111111111111111111111111111111111111111111111111111111111111111111111111111111111111111111111111111111111111111111111111111111111111111111111111111111111111111111111111111111111111111111111111111111111111111111111111111111111111111111111111000000000000000000000000000000000

66666666666666666666666666666666666666666666666666666666666666666666666666666666666666666666666666666666666666666666666666666666666666666666666666666666666666666666666666666666666666666666666666666666666666666666666666666666666666666666666666666666666666666666666666666666666666666666666666666666666666666666000000000000000000000000000000000

00000000000000000000000000000000000000000000000000000000000000000000011111111111111111111111111111111111111111111111111111111111111111111111111111111111111111111111111112222222222222222222222222222222222222222222222222222222222222222222222222222222222222222222222222222333333333333333333333333333333333333333000000000000000000000000000000000

33333333344444444445555555555666666666677777777778888888888999999999900000000001111111111222222222233333333334444444444555555555566666666667777777777888888888899999999990000000000111111111122222222223333333333444444444455555555556666666666777777777788888888889999999999000000000011111111112222222222333333333000000000111111111122222222223333

12345678901234567890123456789012345678901234567890123456789012345678901234567890123456789012345678901234567890123456789012345678901234567890123456789012345678901234567890123456789012345678901234567890123456789012345678901234567890123456789012345678901234567890123456789012345678901234567890123456789012345678123456789012345678901234567890123

REF. ATATAAGCAAGTACATGACCTCTATAGCAGTACATAATACATATAATTATTGACTGTACATAGTACATTATGTCAAATTCATTCTTGATAGTATATCTATTATATATTCCTTACCATTAGATCACGAGCTTAATTACCATGCCGCGTGAAACCAGCAACCCGCTAGGCAGGGATCCCTCTTCTCGCTCCGGGCCCATAAACCGTGGGGGTCGCTATCCAATGAATTTTACCAGGCATCTGGTTCTTTCTTCAGGGCCATCTCATCTAAAACGGTCCATTCTTTCCTCTTAAATAAGACATCTCGATGGACTAATGGCTAATCAGCCCATGCTCACACATAA

Au-It15

FLO 1.1 ..........................A...........................................................................................................TACCATGCCGCGTGAA

FLO 1.1 ..........................A...........................................................................................................

FLO 1.2 ..........................A...........................................................................................................

FLO 1.2 ..........................A...........................................................................................................

FLO 2.1 ..........................A...........................................................................................................

FLO 2.1 ..........................A...........................................................................................................

FLO 2.2 ..........................A...........................................................................................................

FLO 2.2 ..........................A...........................................................................................................

CEA 1.1 ..........................A...C.......................................................................................................

CEA 1.1 ..........................A...........................................................................................................

CEA 1.1 ..........................A.......................................G...................................................................

CEA 1.1 ..........................A...........................................................................................................

CEA 1.1 ..........................A...........................................................................................................

Consensus ATATAAGCAAGTACATGACCTCTATAACAGTACATAATACATATAATTATTGACTGTACATAGTACATTATGTCAAATTCATTCTTGATAGTATATCTATTATATATTCCTTACCATTAGATCACGAGCTTAAT

11111111111111111111111111111111111111111111111111111111111111111111111111111111111111111111111111111111111111111111111111111111111111111111111111111111111111111111111111111111111111111111111111111111111111111111111111111111111111111111111111111111111111111111111111111111111111111111111111111111111111111111000000000000000000000000000000000

66666666666666666666666666666666666666666666666666666666666666666666666666666666666666666666666666666666666666666666666666666666666666666666666666666666666666666666666666666666666666666666666666666666666666666666666666666666666666666666666666666666666666666666666666666666666666666666666666666666666666666666000000000000000000000000000000000

00000000000000000000000000000000000000000000000000000000000000000000011111111111111111111111111111111111111111111111111111111111111111111111111111111111111111111111111112222222222222222222222222222222222222222222222222222222222222222222222222222222222222222222222222222333333333333333333333333333333333333333000000000000000000000000000000000

33333333344444444445555555555666666666677777777778888888888999999999900000000001111111111222222222233333333334444444444555555555566666666667777777777888888888899999999990000000000111111111122222222223333333333444444444455555555556666666666777777777788888888889999999999000000000011111111112222222222333333333000000000111111111122222222223333

12345678901234567890123456789012345678901234567890123456789012345678901234567890123456789012345678901234567890123456789012345678901234567890123456789012345678901234567890123456789012345678901234567890123456789012345678901234567890123456789012345678901234567890123456789012345678901234567890123456789012345678123456789012345678901234567890123

REF. ATATAAGCAAGTACATGACCTCTATAGCAGTACATAATACATATAATTATTGACTGTACATAGTACATTATGTCAAATTCATTCTTGATAGTATATCTATTATATATTCCTTACCATTAGATCACGAGCTTAATTACCATGCCGCGTGAAACCAGCAACCCGCTAGGCAGGGATCCCTCTTCTCGCTCCGGGCCCATAAACCGTGGGGGTCGCTATCCAATGAATTTTACCAGGCATCTGGTTCTTTCTTCAGGGCCATCTCATCTAAAACGGTCCATTCTTTCCTCTTAAATAAGACATCTCGATGGACTAATGGCTAATCAGCCCATGCTCACACATAA

Au-It16

FLO 1.1 TTCCTTACCATTAGATCACGAGC....................................................................................................................TTCTTCAGGGCCATCTCATC

FLO 1.1 ....................................................................................................................

FLO 1.2 ....................................................................................................................

FLO 1.2 ....................................................................................................................

FLO 2.1 ....................................................................................................................

FLO 2.1 ....................................................................................................................

FLO 2.2 ....................................................................................................................

FLO 2.2 ....................................................................................................................

Consensus TTAATTACCATGCCGCGTGAAACCAGCAACCCGCTAGGCAGGGATCCCTCTTCTCGCTCCGGGCCCATAAACCGTGGGGGTCGCTATCCAATGAATTTTACCAGGCATCTGGTTCT

11111111111111111111111111111111111111111111111111111111111111111111111111111111111111111111111111111111111111111111111111111111111111111111111111111111111111111111111111111111111111111111111111111111111111111111111111111111111111111111111111111111111111111111111111111111111111111111111111111111111111111111000000000000000000000000000000000

66666666666666666666666666666666666666666666666666666666666666666666666666666666666666666666666666666666666666666666666666666666666666666666666666666666666666666666666666666666666666666666666666666666666666666666666666666666666666666666666666666666666666666666666666666666666666666666666666666666666666666666000000000000000000000000000000000

00000000000000000000000000000000000000000000000000000000000000000000011111111111111111111111111111111111111111111111111111111111111111111111111111111111111111111111111112222222222222222222222222222222222222222222222222222222222222222222222222222222222222222222222222222333333333333333333333333333333333333333000000000000000000000000000000000

33333333344444444445555555555666666666677777777778888888888999999999900000000001111111111222222222233333333334444444444555555555566666666667777777777888888888899999999990000000000111111111122222222223333333333444444444455555555556666666666777777777788888888889999999999000000000011111111112222222222333333333000000000111111111122222222223333

12345678901234567890123456789012345678901234567890123456789012345678901234567890123456789012345678901234567890123456789012345678901234567890123456789012345678901234567890123456789012345678901234567890123456789012345678901234567890123456789012345678901234567890123456789012345678901234567890123456789012345678123456789012345678901234567890123

REF. ATATAAGCAAGTACATGACCTCTATAGCAGTACATAATACATATAATTATTGACTGTACATAGTACATTATGTCAAATTCATTCTTGATAGTATATCTATTATATATTCCTTACCATTAGATCACGAGCTTAATTACCATGCCGCGTGAAACCAGCAACCCGCTAGGCAGGGATCCCTCTTCTCGCTCCGGGCCCATAAACCGTGGGGGTCGCTATCCAATGAATTTTACCAGGCATCTGGTTCTTTCTTCAGGGCCATCTCATCTAAAACGGTCCATTCTTTCCTCTTAAATAAGACATCTCGATGGACTAATGGCTAATCAGCCCATGCTCACACATAA

Au-It17

FLO 1.1 TTCCTTACCATTAGATCACGAGC....................................................................................................................TTCTTCAGGGCCATCTCATC

FLO 1.1 ....................................................................................................................

FLO 1.1 ....................................................................................................................

FLO 1.2 ....................................................................................................................

FLO 1.2 ....................................................................................................................

FLO 1.2 ....................................................................................................................

FLO 2.1 ....................................................................................................................

FLO 2.1 ....................................................................................................................

FLO 2.2 ....................................................................................................................

FLO 2.2 ....................................................................................................................

Consensus TTAATTACCATGCCGCGTGAAACCAGCAACCCGCTAGGCAGGGATCCCTCTTCTCGCTCCGGGCCCATAAACCGTGGGGGTCGCTATCCAATGAATTTTACCAGGCATCTGGTTCT

11111111111111111111111111111111111111111111111111111111111111111111111111111111111111111111111111111111111111111111111111111111111111111111111111111111111111111111111111111111111111111111111111111111111111111111111111111111111111111111111111111111111111111111111111111111111111111111111111111111111111111111000000000000000000000000000000000

66666666666666666666666666666666666666666666666666666666666666666666666666666666666666666666666666666666666666666666666666666666666666666666666666666666666666666666666666666666666666666666666666666666666666666666666666666666666666666666666666666666666666666666666666666666666666666666666666666666666666666666000000000000000000000000000000000

00000000000000000000000000000000000000000000000000000000000000000000011111111111111111111111111111111111111111111111111111111111111111111111111111111111111111111111111112222222222222222222222222222222222222222222222222222222222222222222222222222222222222222222222222222333333333333333333333333333333333333333000000000000000000000000000000000

33333333344444444445555555555666666666677777777778888888888999999999900000000001111111111222222222233333333334444444444555555555566666666667777777777888888888899999999990000000000111111111122222222223333333333444444444455555555556666666666777777777788888888889999999999000000000011111111112222222222333333333000000000111111111122222222223333

12345678901234567890123456789012345678901234567890123456789012345678901234567890123456789012345678901234567890123456789012345678901234567890123456789012345678901234567890123456789012345678901234567890123456789012345678901234567890123456789012345678901234567890123456789012345678901234567890123456789012345678123456789012345678901234567890123

REF. ATATAAGCAAGTACATGACCTCTATAGCAGTACATAATACATATAATTATTGACTGTACATAGTACATTATGTCAAATTCATTCTTGATAGTATATCTATTATATATTCCTTACCATTAGATCACGAGCTTAATTACCATGCCGCGTGAAACCAGCAACCCGCTAGGCAGGGATCCCTCTTCTCGCTCCGGGCCCATAAACCGTGGGGGTCGCTATCCAATGAATTTTACCAGGCATCTGGTTCTTTCTTCAGGGCCATCTCATCTAAAACGGTCCATTCTTTCCTCTTAAATAAGACATCTCGATGGACTAATGGCTAATCAGCCCATGCTCACACATAA

Au-It18

FLO 1.1 TTCCTTACCATTAGATCACGAGC....................................................................................................................TTCTTCAGGGCCATCTCATC

FLO 1.1 ...............................T......A......T.......................T..............................................

FLO 1.1 ....................................................................................................................

FLO 1.1 ....................................................................................................................

FLO 1.2 ....................................................................................................................

FLO 1.2 ....................................................................................................................

FLO 1.2 ....................................................................................................................

FLO 2.1 ....................................................................................................................

FLO 2.1 ....................................................................................................................

FLO 2.2 ....................................................................................................................

FLO 2.2 ....................................................................................................................

Consensus TTAATTACCATGCCGCGTGAAACCAGCAACCCGCTAGGCAGGGATCCCTCTTCTCGCTCCGGGCCCATAAACCGTGGGGGTCGCTATCCAATGAATTTTACCAGGCATCTGGTTCT

11111111111111111111111111111111111111111111111111111111111111111111111111111111111111111111111111111111111111111111111111111111111111111111111111111111111111111111111111111111111111111111111111111111111111111111111111111111111111111111111111111111111111111111111111111111111111111111111111111111111111111111000000000000000000000000000000000

66666666666666666666666666666666666666666666666666666666666666666666666666666666666666666666666666666666666666666666666666666666666666666666666666666666666666666666666666666666666666666666666666666666666666666666666666666666666666666666666666666666666666666666666666666666666666666666666666666666666666666666000000000000000000000000000000000

00000000000000000000000000000000000000000000000000000000000000000000011111111111111111111111111111111111111111111111111111111111111111111111111111111111111111111111111112222222222222222222222222222222222222222222222222222222222222222222222222222222222222222222222222222333333333333333333333333333333333333333000000000000000000000000000000000

33333333344444444445555555555666666666677777777778888888888999999999900000000001111111111222222222233333333334444444444555555555566666666667777777777888888888899999999990000000000111111111122222222223333333333444444444455555555556666666666777777777788888888889999999999000000000011111111112222222222333333333000000000111111111122222222223333

12345678901234567890123456789012345678901234567890123456789012345678901234567890123456789012345678901234567890123456789012345678901234567890123456789012345678901234567890123456789012345678901234567890123456789012345678901234567890123456789012345678901234567890123456789012345678901234567890123456789012345678123456789012345678901234567890123

REF. ATATAAGCAAGTACATGACCTCTATAGCAGTACATAATACATATAATTATTGACTGTACATAGTACATTATGTCAAATTCATTCTTGATAGTATATCTATTATATATTCCTTACCATTAGATCACGAGCTTAATTACCATGCCGCGTGAAACCAGCAACCCGCTAGGCAGGGATCCCTCTTCTCGCTCCGGGCCCATAAACCGTGGGGGTCGCTATCCAATGAATTTTACCAGGCATCTGGTTCTTTCTTCAGGGCCATCTCATCTAAAACGGTCCATTCTTTCCTCTTAAATAAGACATCTCGATGGACTAATGGCTAATCAGCCCATGCTCACACATAA

Au-It19

FLO 1.1 TTCCTTACCATTAGATCACGAGC....................................................................................................................TTCTTCAGGGCCATCTCATC

FLO 1.1 ....................................................................................................................

FLO 1.1 ....................................................................................................................

FLO 1.1 ....................................................................................................................

FLO 1.2 ....................................................................................................................

FLO 1.2 ....................................................................................................................

FLO 1.2 ....................................................................................................................

FLO 2.1 ....................................................................................................................

FLO 2.1 ................................................C...................................................................

FLO 2.2 ....................................................................................................................

FLO 2.2 ....................................................................................................................

Consensus TTAATTACCATGCCGCGTGAAACCAGCAACCCGCTAGGCAGGGATCCCTCTTCTCGCTCCGGGCCCATAAACCGTGGGGGTCGCTATCCAATGAATTTTACCAGGCATCTGGTTCT
