# Supplementary material for: Population dynamic of the extinct European aurochs: genetic evidence of a north-south differentiation pattern and no evidence of post-glacial expansion
Source: BMC Evol Biol. 2010 Mar 26;10:83. doi: 10.1186/1471-2148-10-83 (PMC2858146; doi:10.1186/1471-2148-10-83)
Supplement: Additional file 3 — Table S3. The consensus sequence for each sample. [file 1471-2148-10-83-S3.DOC]

Table S3. The mitochondrial HVR-I sequences. The *Bos taurus* reference sequence (Anderson et al., 1982) is also included. Diagnostic sites (Troy et a., 2001) are used to tentatively attribute each individual to mtDNA haplogroups (see column 2); dubious (multiple)attributions are indicated by /. The length of the successfully typed sequence is reported in the third column.

11111111111111111111111111111111111111111111111111111111111111111111111111111111111111111111111111111111111111111111111111111111111111111111111111111111111111111111111111111111111111111111111111111111111111111111111111111111111111111111111111111111111111111111111111111111111111111111111111111111111111111111000000000000000000000000000000000

66666666666666666666666666666666666666666666666666666666666666666666666666666666666666666666666666666666666666666666666666666666666666666666666666666666666666666666666666666666666666666666666666666666666666666666666666666666666666666666666666666666666666666666666666666666666666666666666666666666666666666666000000000000000000000000000000000

00000000000000000000000000000000000000000000000000000000000000000000011111111111111111111111111111111111111111111111111111111111111111111111111111111111111111111111111112222222222222222222222222222222222222222222222222222222222222222222222222222222222222222222222222222333333333333333333333333333333333333333000000000000000000000000000000000

33333333344444444445555555555666666666677777777778888888888999999999900000000001111111111222222222233333333334444444444555555555566666666667777777777888888888899999999990000000000111111111122222222223333333333444444444455555555556666666666777777777788888888889999999999000000000011111111112222222222333333333000000000111111111122222222223333

12345678901234567890123456789012345678901234567890123456789012345678901234567890123456789012345678901234567890123456789012345678901234567890123456789012345678901234567890123456789012345678901234567890123456789012345678901234567890123456789012345678901234567890123456789012345678901234567890123456789012345678123456789012345678901234567890123

REF T3 341 ATATAAGCAAGTACATGACCTCTATAGCAGTACATAATACATATAATTATTGACTGTACATAGTACATTATGTCAAATTCATTCTTGATAGTATATCTATTATATATTCCTTACCATTAGATCACGAGCTTAATTACCATGCCGCGTGAAACCAGCAACCCGCTAGGCAGGGATCCCTCTTCTCGCTCCGGGCCCATAAACCGTGGGGGTCGCTATCCAATGAATTTTACCAGGCATCTGGTTCTTTCTTCAGGGCCATCTCATCTAAAACGGTCCATTCTTTCCTCTTAAATAAGACATCTCGATGGACTAATGGCTAATCAGCCCATGCTCACACATAA

Au-It6 T3 245 .....................................................................................................................................................................................................................................................------------------------------------------------------------------------------------------------

Au-It7 T/T3 134 ......................................................................................................................................---------------------------------------------------------------------------------------------------------------------------------------------------------------------------------------------------------------

Au-It8 T/T3 238 ......................................................................................................................................-------------------------------------------------------------------------------------------------------........................................................................................................

Au-It9 T/T3 134 ......................................................................................................................................---------------------------------------------------------------------------------------------------------------------------------------------------------------------------------------------------------------

Au-It10 T2 134 .........................CT..........................................................................................................---------------------------------------------------------------------------------------------------------------------------------------------------------------------------------------------------------------

Au-It11 T/T3 134 ................................................................................................T.....................................---------------------------------------------------------------------------------------------------------------------------------------------------------------------------------------------------------------

Au-It12 T3 341 .....................................................................................................................................................................................................................................................................................................................................................

Au-It13 P 134 ..........A.......T.C......T...............C..........C....................................C..........................................---------------------------------------------------------------------------------------------------------------------------------------------------------------------------------------------------------------

Au-It14 P 245 ..................T.C......T...............C..........C....................................C......................................................................T......A......T.......................T............................................------------------------------------------------------------------------------------------------

Au-It15 T/T3 134 ..........................A...........................................................................................................---------------------------------------------------------------------------------------------------------------------------------------------------------------------------------------------------------------

Au-It16 T3 116 ---------------------------------------------------------------------------------------------------------------------------------....................................................................................................................------------------------------------------------------------------------------------------------

Au-It17 T3 116 ---------------------------------------------------------------------------------------------------------------------------------....................................................................................................................------------------------------------------------------------------------------------------------

Au_It18 T3 116 ---------------------------------------------------------------------------------------------------------------------------------....................................................................................................................------------------------------------------------------------------------------------------------

Au-It19 T3 116 ---------------------------------------------------------------------------------------------------------------------------------....................................................................................................................------------------------------------------------------------------------------------------------

References

Anderson, S., M. H. de Bruijn, A. R. Coulson, I. C. Eperon, F. Sanger, and I. G. Young. 1982. Complete sequence of bovine mitochondrial DNA. Conserved features of the mammalian mitochondrial genome. J Mol Biol 156:683-717.

Troy CS, MacHugh DE, Bailey JF, Magee DA, Loftus RT, Cunningham P, Chamberlain AT, Sykes BC, Bradley DG. 2001. Genetic evidence for Near-Eastern origins of European cattle. Nature. 410:1088-1091
